# Indole prevents *Escherichia coli* cell division by modulating membrane potential

**DOI:** 10.1016/j.bbamem.2012.02.022

**Published:** 2012-07

**Authors:** Catalin Chimerel, Christopher M. Field, Silvia Piñero-Fernandez, Ulrich F. Keyser, David K. Summers

**Affiliations:** a Department of Genetics, University of Cambridge, Downing Street, Cambridge CB2 3EH, United Kingdom; b Cavendish Laboratory University of Cambridge, Department of Physics, JJ Thomson Avenue, Cambridge, CB3 0HE, United Kingdom

**Keywords:** Membrane potential, Proton ionophore, Indole, Bacteria, Cell division, *Escherichia coli*

## Abstract

Indole is a bacterial signalling molecule that blocks *E. coli* cell division at concentrations of 3–5 mM. We have shown that indole is a proton ionophore and that this activity is key to the inhibition of division. By reducing the electrochemical potential across the cytoplasmic membrane of *E. coli*, indole deactivates MinCD oscillation and prevents formation of the FtsZ ring that is a prerequisite for division. This is the first example of a natural ionophore regulating a key biological process. Our findings have implications for our understanding of membrane biology, bacterial cell cycle control and potentially for the design of antibiotics that target the cell membrane.

## Highlights

► Indole induces charge transport across reconstituted lipid membranes. ► Indole reduces the *E. coli* membrane potential. ► Membrane potential is required for the formation of the macromolecular divisome. ► Indole blocks the formation of the macromolecular divisome. ► Indole reversibly blocks *Escherichia coli* cell division.

## 1 Introduction

Indole is an aromatic heterocycle produced by over 85 species of Gram-positive and Gram-negative bacteria with multiple and diverse roles in bacterial signalling [1]. In addition to regulating the transition from exponential to stationary phase [2], biofilm formation [3] and responses to virulence and stress [4], it has also been shown to mediate signalling between enteric bacteria and their mammalian host [5]. Recently indole has been found to inhibit *Escherichia coli* cell division as part of a cell cycle checkpoint triggered by the accumulation of plasmid dimers [6,7]. Plasmid dimers produce a regulatory RNA (Rcd) that stimulates indole synthesis by the enzyme tryptophanase, but the mechanism by which indole prevents cell division was unknown. A link between membrane potential and cell division has been reported previously [8] and ionophores such as carbonyl cyanide m-chloro phenyl hydrazine (CCCP) block cell division by dissipating the proton motive force (PMF) [9]. Here we show that a previously unrecognised property of indole, its action as an ionophore, decreases the PMF across the *E. coli* cytoplasmic membrane and hence inhibits cell division.

## 2 Materials and methods

### 2.1 The expression of fluorescent proteins

*E. coli* BW25113Δ*tnaA* was obtained from the Keio collection [10] and *E. coli* W3110Δ*tnaA* [7] was derived from this strain by P1 transduction. Cells were cultured in Luria Bertani (LB) liquid medium at 37 °C. Kanamycin (30 μg ml^− 1^) was added to overnight cultures. Overnight cultures were diluted for use to OD_600_ = 0.03 (approx.) in 20 ml of LB. When required, indole (in ethanol) or CCCP (in DMSO) were added when the culture reached OD_600_ = 0.1–0.2. The final concentration of ethanol in the samples containing indole, and in the indole-free control, was 1.0% (v/v). The maximum final concentration of DMSO was 0.1% (v/v). Plasmids used for the expression of fluorescent proteins were: pCP8 (also known as pWX3∆; P_ftsKi_-*ftsZ*-*cfp* [11]), pHJS101 (P_ara_-*sf*-*gfp*-*ftsA* [8]) and pFX9 (P_lac_-*gfp*-*minD minE* [12]).

### 2.2 Fluorescence microscopy

**Fig. 1:**
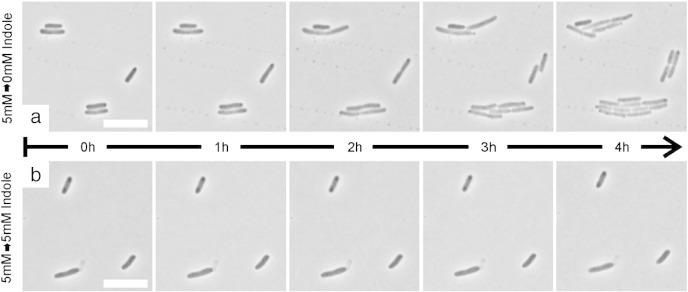
Indole reversibly inhibits growth and division of *E. coli. E. coli* BW25113 *ΔtnaA* growing in LB at 37 °C was incubated with 5 mM indole for 2 h. Culture samples were immobilised at room temperature on agarose-coated slides without indole (panel a) or containing 5 mM indole (panel b). Cells were observed by phase contrast microscopy over the next 4 h. Size bar represents 10 μM.

**Fig. 2:**
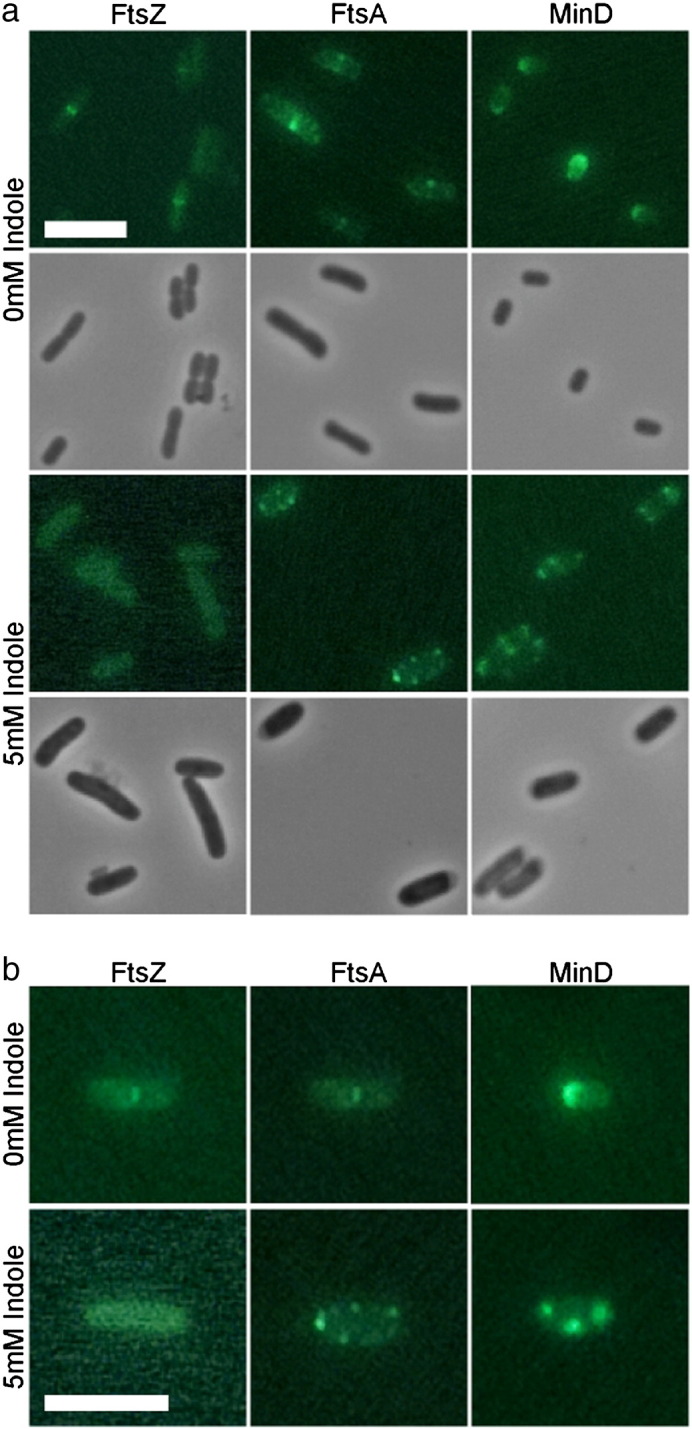
Indole-dependent delocalisation of FtsZ, FtsA and MinD. Panel (a) shows paired images (phase contrast and fluorescence) in the presence and absence of indole (5 mM). Panel (b) shows enlargements of representative cells (fluorescence only). Fluorescent proteins were expressed from plasmids pCP8 (PftsKi-FtsZ-CFP), pHJS101 (P_ara_-sfGFP-FtsA) and pFX9 (Plac-GFP-MinD MinE). Size bar represents 5 μm.

For microscopy, cells were immobilised on a thin film of 1% agarose in phosphate buffered saline (Fig. 2) or minimal salts with 1% glucose (Fig. 1), containing indole (5 mM) when required. A Nikon Eclipse 80i microscope equipped with a 100× CFI Plan Fluor objective was used for phase contrast visualisation. NIS-elements F 3.0 software (Nikon) was used for image acquisition.

### 2.3 Electrophysiology

Artificial lipid bilayers made of *E. coli* total lipid extract (Avanti Polar Lipid) were reconstituted in the round aperture (90 μm diameter) of a Teflon foil, using the Montal–Mueller technique [13,14]. A 1% (v/v) hexadecane solution was used to paint the Teflon foil before bilayer formation. The chambers encompassing the Teflon foil were first filled with aqueous solution (100 mM KCl, pH 7) then 5 μl *E. coli* total lipid extract (5 mg ml^− 1^ in pentane) was spread on the surface of the water in the compartments to allow the lipid bilayer to form. The pH of the solutions was controlled using 15 mM potassium phosphate (PB) in acidic or basic form (KH_2_PO_4_/K_2_HPO_4_, respectively). To establish a pH gradient, the pH was adjusted using 10% HCl or KOH. The current–voltage (IV) characteristics of the lipid bilayer were measured in the range ± 50 mV. The applied voltage was varied in steps of 10 mV and the current was recorded for 10 s. A Gaussian fit was made to the histogram of the current trace and the average value of the current determined. A linear fit was applied to the IV curve and the ionic conductance through each bilayer is given by the gradient of the linear fit. The capacitance of the lipid bilayer was determined prior to the measurement of the IV characteristic. By convention the chamber of the lipid bilayer where the ground electrode of the amplifier is located was denoted as *cis* and the one containing the live electrode was denoted as *trans*.

### 2.4 Flow cytometry

For *in vivo* measurement of membrane polarity W3110*ΔtnaA* cells were grown in broth culture to OD_600_ = 0.2 (approx.). When required, indole (in ethanol) or CCCP (in DMSO) were added to samples before the addition of 10 μM oxonol VI (in ethanol). The final concentration of ethanol in the samples containing indole, and in the indole-free control, was 1.5% (v/v). The maximum final concentration of DMSO was 0.1% (v/v). Samples were incubated for 10 min before the addition of formaldehyde (1% w/v). Flow cytometry was performed on a Cytek DxP8 FACScan (Cytek). Fluorescence was excited by a 100 mW laser at 561 nm and measured through a 615 nm emission filter of 25 nm width. For each sample, 50,000 events were collected at a rate between 1000 and 2000 events *per* second. Data were collected and analysed using FlowJo 7.6.4 (Tree Star Inc.). Each sample was first gated by front and side-scatter values to exclude the 3–5% of cells furthest from the median. The remainder were gated by fluorescence value to remove the 1–2% of the population that were obviously undyed (identifiable by their negative fluorescence value). In the data presented, a further 1–2% of cells are likely to be undyed but show a low positive fluorescence due to instrument noise.

## 3 Results

When 5 mM indole is added to a broth culture of *E. coli*, cell division is abolished immediately. The non-dividing cells continue to grow slowly for up to 2 h, approximately doubling in size [7]. To investigate whether indole inhibition of cell division is reversible, 5 mM indole was added to *E. coli* BW25113 *ΔtnaA* growing exponentially at 37 °C (extra-cellular and intra-cellular indole concentrations equilibrate rapidly in this strain [7]). After a further 2 h incubation, culture samples were transferred to agarose-coated slides, with or without indole (5 mM), for microscopic observation. Cells on indole-free agarose (Fig. 1a) resumed growth and division after a lag of approximately 1 h, while on indole-containing agarose (Fig. 1b) cells neither grew nor divided for at least 4 h.

Bacterial cell division begins with the assembly of the macromolecular divisome complex. In *E. coli* the tubulin homologue FtsZ [15], assisted by FtsA [16], polymerises into a ring structure at mid-cell, defining the eventual site of division. We investigated the effect of indole on the localisation of fluorescently-tagged FtsZ and FtsA in *E. coli* BW25113 *ΔtnaA*. Indole (5 mM) was added to a culture of exponentially-growing cells and incubation was continued at 37 °C for 2 h before culture samples were immobilised on agarose-coated slides (5 mM indole) for observation. Parallel observations were made of a control culture without indole. In the absence of indole FtsZ localised at the mid-point of cells as the cell cycle progressed. However, in cells that had been exposed to indole, FtsZ fluorescence was distributed uniformly throughout the cytoplasm (Fig. 2; Supplementary Fig. 1). The septum-associated protein, FtsA, normally localises to the mid-cell in an FtsZ-dependent manner [17]. In the presence of indole FtsA became delocalised from the mid-point and was present in foci distributed throughout the cell (Fig. 2; Supplementary Fig. 2).

The Min site-selection system is a primary determinant of FtsZ spatial distribution [18]. We therefore explored the effect of indole on the localisation of GFP-MinD. Samples from a culture of *E. coli* BW25113 *ΔtnaA* growing exponentially at 37 °C were immobilised on agarose-coated slides, with or without indole (5 mM). In the absence of indole, MinD oscillated between the cell poles with a period of approximately 20 s. On indole-containing slides MinD no longer oscillated but was localised in foci distributed throughout the cell (Fig. 2, Supplementary Fig. 3, Supplementary movies 1–3). The response of MinD to indole was complete within the 2 min required to immobilise cells for observation.

While speculating on the mechanism by which indole inhibits *E. coli* cell division, we were intrigued by a report that division of both *E. coli* and *B. subtilis* requires the maintenance of an electrical potential difference across the cytoplasmic membrane [8]. Dissipation of the electrical potential by the proton ionophore CCCP [19] stops MinD oscillation, disrupting FtsZ ring formation at mid-cell and preventing division. It has been shown that maintenance of the electrical potential is required for efficient binding of the C-terminal amphipathic helix of MinD to the lipid membrane [8]. We also noted that 30 years ago indole had been found to uncouple mitochondrial oxidative phosphorylation [20]. Both mitochondrial uncoupling and the *E. coli* cell division block could be explained if indole acts as an ionophore, conducting ions across a lipid membrane.

**Fig. 3:**
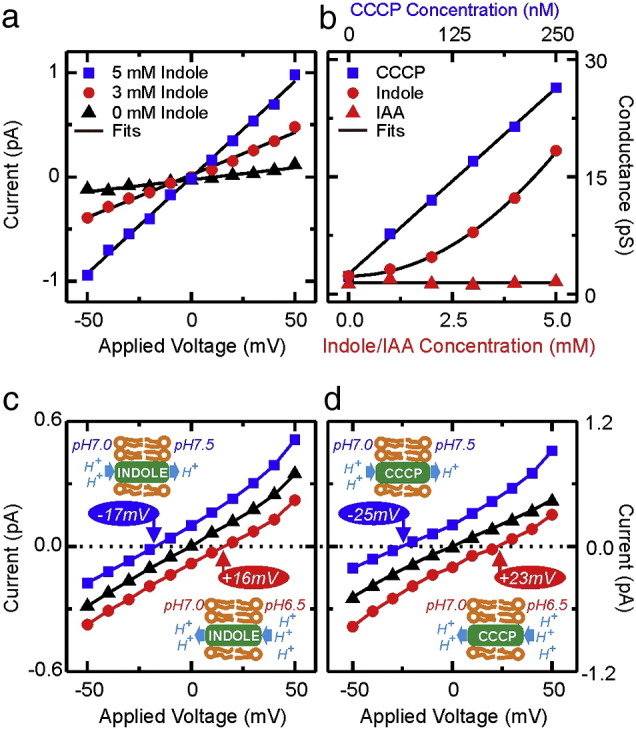
Indole facilitates the transport of ions across artificially reconstituted lipid membranes. Panel a: The IV characteristics of an *E. coli* lipid membrane (100 mM KCl, pH 7.0 in PB) in the presence of indole (*cis* and *trans* chambers). Lines represent linear fits to the data. Panel b: The ionic conductance, calculated from the slope of the IV characteristic, as a function of the concentration of cyanide-m-chlorophenylhydrazone (CCCP), indole and indole 3-acetic acid (IAA). Representative titration experiments for CCCP, indole and IAA are shown. A linear fit is shown for the CCCP and IAA data while a quadratic fit (Eq. (1)) is shown for indole. Each experiment was repeated at least 4 times (Supplementary Fig. 4). Panels c and d: The effect of a pH gradient on the IV characteristics of an *E. coli* lipid membrane in the presence of 2.5 mM indole (panel c) or 50 nM CCCP (panel d). The *trans* chamber is held at pH 7.0 while the pH in the *cis* chamber is varied. Symbols denote the pH on either side of the membrane (−−) 7.0-7.0, (−−) 7.0-6.5, (−−) 7.0–7.5. Experiments were conducted at room temperature (21 °C).

A standard electrophysiology approach was used to investigate the effect of indole on a bilayer reconstituted from *E. coli* total lipid extract. The apparatus consisted of two chambers (*cis* and *trans*) each containing buffer (pH 7.0) and an electrode. The chambers were connected by an aperture (diameter 90 μm), across which the lipid bilayer was established. The ionic conductance of the bilayer, determined from the slope of the current–voltage (IV) curves, was plotted against the indole concentration (Fig. 3a, b; Supplementary Fig. 4). Increasing the indole concentration over the range 0–5 mM changed the bilayer conductance from ~ 2 pS to ~ 18 pS. CCCP was used as a positive control and, over the range 0–250 nM, it increased conductance from ~ 2 pS to ~ 27 pS. As a negative control, ionic conductance was measured in the presence of indole-3-acetic acid (IAA). IAA had no effect on conductance over the range 0–5 mM. The effect of indole on bilayer conductance was reversible, confirming that membrane integrity was not affected by indole treatment (Supplementary Fig. 5).

Curve fitting to the conductance–concentration plots (Fig. 3b) showed that while membrane conductance increased linearly with the CCCP concentration [21,22], the dependence on the indole concentration was quadratic. This type of dependence has been discussed previously [23–26] and is explained by a model in which shuttling of the charged species depends upon the formation of a carrier dimer. Here the carrier is indole, denoted as [IH], and we speculate that the dimer shuttling charge across the lipid membrane might be [I_2_H]^-^. The ionic conductance of the lipid bilayer as function of indole concentration is then predicted by:

(1) $$K\left(c\right)=\alpha \;c^{2}+K_{0}$$

where *c* is the indole concentration, *K*(*c*) is the ionic conductance through the lipid bilayer as a function of indole concentration, *K*_0_ is the leakage conductance through the lipid bilayer in the absence of indole and α is a proportionality constant independent of the indole concentration. When the dependence of conductance on the indole concentration (Fig. 3b) is fitted with Eq. (1), an experimental fitting parameter of *R*^2^ = 0.99904 is obtained.

In the initial experiments (Fig. 3a, b) the pH of both chambers was 7.0. In subsequent experiments the pH of the *trans* chamber was retained at 7.0 while the pH in the *cis* chamber was either increased to 7.5 by addition of KOH or reduced to 6.5 by the addition of HCl. The membrane IV characteristic was determined in the presence of CCCP (50 nM) or indole (2.5 mM) (Fig. 3c, d). In both cases when a pH gradient existed across the membrane a current was detected in the absence of an applied voltage. Formally this could be due to positive charges moving from low pH to the high pH, or to negative charges moving in the opposite direction. A voltage (− 50 mV to + 50 mV) was applied across the lipid bilayer and the value required to reduce the current to zero was noted. In the presence of indole an applied voltage of 33 mV per pH unit was required, while in the presence of CCCP the average voltage was 48 mV per pH unit (Fig. 3c, d).

The theoretical model that predicts the quadratic dependence of the lipid bilayer conductance on the indole concentration [23–26] gives the following relationship between the pH gradient and the voltage required to abolish current flow:

(2) $$V=\frac{RT}{F}\ln\frac{{\left[H^{+}\right]}^{1}}{{\left[H^{+}\right]}^{2}}$$

where *V* is the trans-membrane voltage due to the pH gradient, *R* is the gas constant, *T* is absolute temperature, *F* is the Faraday constant and [H^+^]^1^, [H^+^]^2^ are the proton concentrations on the *cis* and *trans* sides of the electrophysiology chamber.

Eq. (2) predicts a trans-membrane voltage of 58.4 mV per pH unit at 21 °C, irrespective of the ionophore. There is a significant difference between the theoretical prediction and the measured values for indole and CCCP (33 and 48 mV per pH unit, respectively). This is because, in the presence of 2.5 mM indole or 50 nM CCCP the leakage ionic conductance of the lipid bilayer, representing the ions that pass the lipid bilayer not being carried by the ionophore, is not negligible compared to the ionophore induced conductance [19]. In brief, the leakage conductance creates a voltage divider and thus reduces the measured trans-membrane voltage (*V*_m_) as given by [19]:

(3) $$V_{\text{m}}≅V\frac{K(c)-K_{0}}{K(c)}$$

Here the terms of the equation are the same as previously defined in Eq. (1) and Eq. (2).

In the presence of 2.5 mM indole (Fig. 3c, d) the lipid bilayer conductance is 6.0 pS while the leakage conductance in the absence of the ionophore is 2.1 pS. Thus Eq. (3) predicts a measured trans-membrane potential of 38 mV per pH unit that is close to the measured experimental value of 33 mV per pH unit. In the case of 50 nM CCCP, the membrane conductance is 9.0 pS while the leakage conductance 1.6 pS. Thus Eq. (3) predicts a trans-membrane voltage of 48 mV per pH unit in very good agreement with the experimentally measured value.

**Fig. 4:**
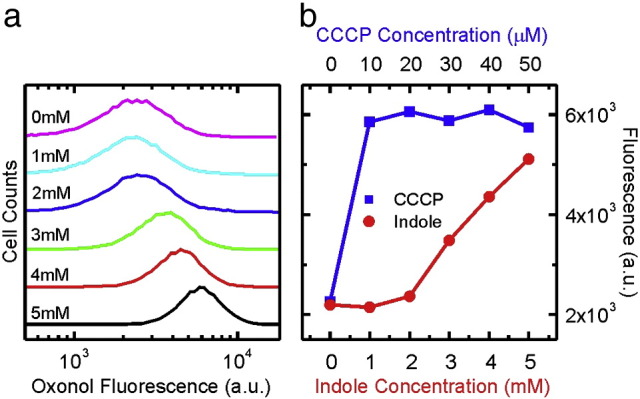
Indole depolarises the *E. coli* membrane in a concentration-dependent manner. *E. coli* W3110ΔtnaA was treated with the lipophilic dye Oxonol VI in the presence of indole (0–5 mM). Cells were observed for fluorescence by flow cytometry (panel a) and the median value of each sample plotted against indole concentration (panel b). Data presented are from a single representative experiment (see Supplementary Material Fig. 6 for biological repeats).

To ascertain whether indole acts as an ionophore *in vivo*, we used flow cytometry to observe the effect of indole on binding of the fluorescent dye oxonol VI to *E. coli* cells. Oxonol VI is an anionic dye that binds lipid membranes, with a greater affinity for depolarised membranes [27]. Thus permeabilisation of the cytoplasmic membrane by an ionophore should increase oxonol VI binding and cell fluorescence. The fluorescence intensity increased noticeably at 3 mM indole and above (Fig. 4a and Supplementary Fig. 6). Cells treated with 5 mM indole showed a 2.5-fold increase in median fluorescence intensity, compared with untreated cells. This was similar to the effect of CCCP (10–50 μM) that was used as a positive control (Fig. 4b).

## 4 Discussion

In summary, our data suggest that the action of indole as an ionophore is responsible for its inhibition of cell division. Depolarisation of the cytoplasmic membrane inactivates the Min site selection system, preventing the localisation of FtsZ and the formation of the divisome complex. We have noted previously [6] that although indole has little effect on *E. coli* up to 2–3 mM, severe inhibition of growth and cell division appears suddenly over the 3–5 mM range. The non-linearity of this response may be explained by the quadratic dependence of membrane conductance on the indole concentration.

It is known that indole is capable of diffusing through a lipid membrane [7] but here we show that the membrane itself is a target of indole action. Biological ionophores are well known but they are normally toxic to cells. Thus the plasmid-encoded colicins A, E1, Ia, Ib, and K form ion-permeable channels in the bacterial cytoplasmic membrane leading to cell death [28]. Similarly the antibiotics nigericin [29] and valinomycin [30], produced by Streptomyces species, facilitate ion transport across the cytoplasmic membrane, dissipating the pH and electrical potentials, respectively. The novelty of indole as an ionophore is that it directly benefits the producer cell.

Cell cycle arrest is a common response of eukaryotic [31] and prokaryotic [32] cells to DNA damage. Circular DNA molecules, common in bacteria, suffer a unique form of recombinational damage to which their linear counterparts are immune. While the recombination between linear replicons does not compromise their independence, recombination between DNA circles forms a dimer in which they are joined covalently. One response to recombinational damage of circular plasmids in *E. coli* is the arrest of cell division [33] and this has been shown to correlate with the up-regulation of indole production [6].

Indole increases the survival of bacteria under antibiotic stress, and indole production is stimulated by exposure to antibiotics, including ampicillin and kanamycin [34]. It is generally accepted that indole protects bacteria against antibiotics by inducing multidrug exporter genes [35]. However, the ionophore property of indole may provide an additional protection mechanism. The uptake of aminoglycoside antibiotics (e.g. tetracycline) is energised by the PMF across the cytoplasmic membrane [36,37]. Reduction of the PMF by indole should prevent, or at least reduce, the uptake of antibiotics that use this mechanism. It is also possible that intracellular indole concentrations of several millimolars have biologically significant effects on enzyme activity and protein–protein interactions. This possibility is under active investigation in the authors' laboratories.

A clear distinction between indole and CCCP is that the latter is effective as an ionophore at a 10^4^-fold lower concentration than indole. One millimolar indole was required to increase membrane conductance by an average of 47% (Supplementary Fig. 4). Typical supernatant concentrations of indole in stationary phase culture of *E. coli* are only 0.3–0.7 mM [6,38] but there is reason to believe that millimolar concentrations of indole may be a reality in the natural environment, especially under stress conditions. It has been reported that *E. coli* in broth culture produces elevated levels of indole in response to antibiotic or heat stress [34] and an intracellular indole concentration of 1.7 mM has been reported in a strain where plasmid multimerisation is up-regulated [6]. Typically experiments measuring indole production are performed in broth culture when secreted indole is rapidly dispersed and diluted. In diffusion-limited environments such as the mammalian gut [5], or where bacteria grow as colonies or as biofilms on a solid substrate, secreted indole will remain associated with the producer cells giving higher local concentrations. Furthermore, we would expect quantitative variation in the effect of indole on membranes with differing lipid composition and it is interesting to note that while more than 3 mM indole is required to inhibit growth and division of laboratory strains of *E. coli*, as little as 0.5 mM has been shown to uncouple oxidative phosphorylation in rat liver mitochondria [20] and 1 mM indole induces potentially beneficial changes in human intestinal epithelial cells *in vitro* [5].

Finally, one of the most remarkable aspects of indole signalling is its universality. Bacteria use indole to signal to themselves, to other bacterial species and even to their mammalian host [1]. Our discovery that indole is an ionophore provides a signalling mechanism that is applicable to all living cells.

## Appendix A Supplementary data

Supplementary materials

Video S1 The movie shows the oscillation of MinD between the poles of *E. coli* in the absence of indole. Fluorescent protein was expressed from plasmid pFX9 (Plac-GFP-MinD MinE) in all three supplementary movies. The movie represents 261.2 s (real time) and is composed of 76 frames. The scale bar represents 5 μm.

Video S2 The movie shows the absence of MinD oscillation between the poles of the *E. coli* in the presence of 5 mM indole. The movie represents 130.5 s (real time) and is composed of 34 frames. The scale bar represents 5 μm.

Video S3 The movie, like supplementary movie 2, shows the absence of MinD oscillation between the poles of the *E. coli* in the presence of 5 mM indole. The movie represents 87 s (real time) and is composed of 23 frames. The scale bar represents 5 μm.
