# Supplementary material for: Indole prevents Escherichia coli cell division by modulating membrane potential
Source: Biochim Biophys Acta. 2012 Jul;1818(7):1590–4. doi: 10.1016/j.bbamem.2012.02.022 (PMC3793866; doi:10.1016/j.bbamem.2012.02.022)
Supplement: Supplementary file 1 — Supplementary materials [file mmc1.doc]

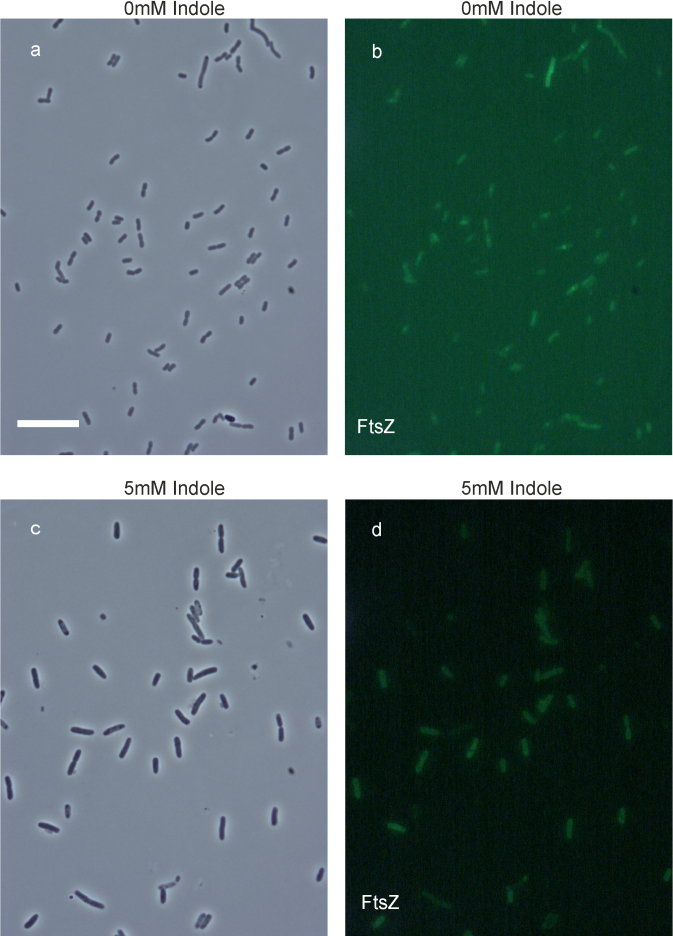


**Figure 1. Indole-dependent delocalization of FtsZ.** The panels show paired images (phase contrast and fluorescence) of untreated cells (a, b) or cells treated with 5 mM indole (c, d). Size bar represents 10µm. About 27% of the cells show the formation of the fluorescent ring in the absence of indole but none of the cells shows ring formation in the presence of 5mM indole.


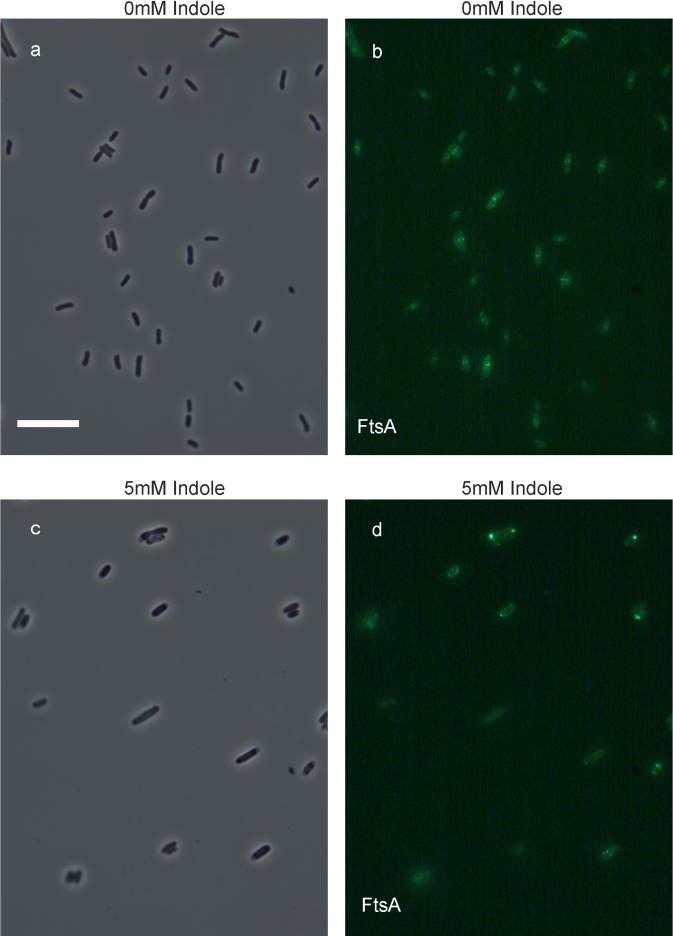


**Figure 2. Indole-dependent delocalization of FtsA.** The panels show paired images (phase contrast and fluorescence) of untreated cells (a, b) or cells treated with 5 mM indole (c, d). Size bar represents 10µm. About 69% of the cells show the formation of the fluorescent ring in the absence of indole but none of the cells shows ring formation in the presence of 5mM indole.


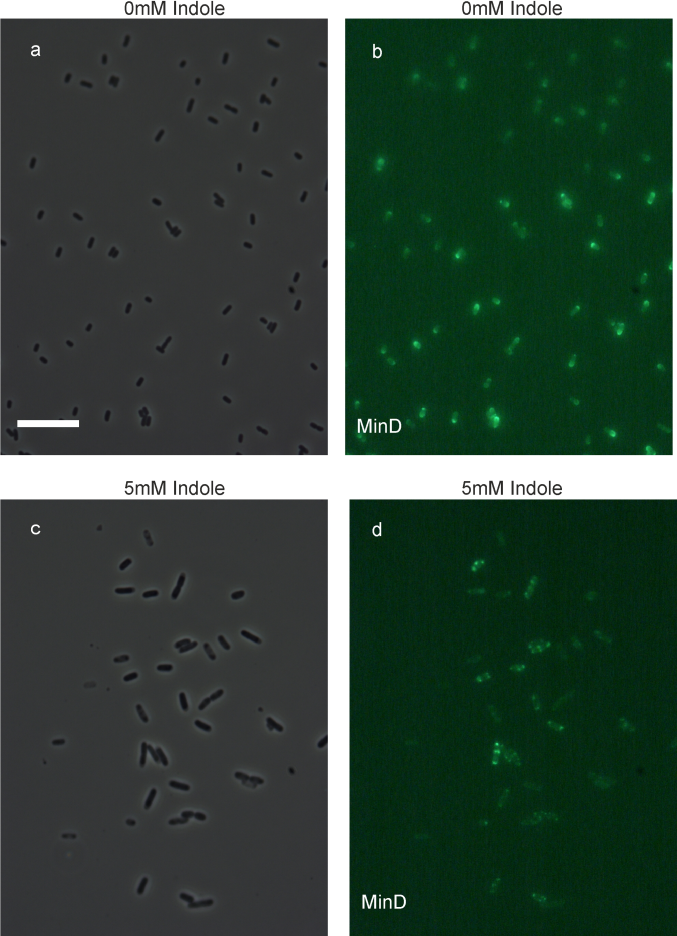


**Figure 3. Indole-dependent delocalization of MinD.** The panels show paired images (phase contrast and fluorescence) of untreated cells (a, b) or cells treated with 5 mM indole (c, d). Size bar represents 10µm. More than 90% of the cells show MinD oscillation between the cell poles, but no oscillation was observed in the presence of 5mM indole.


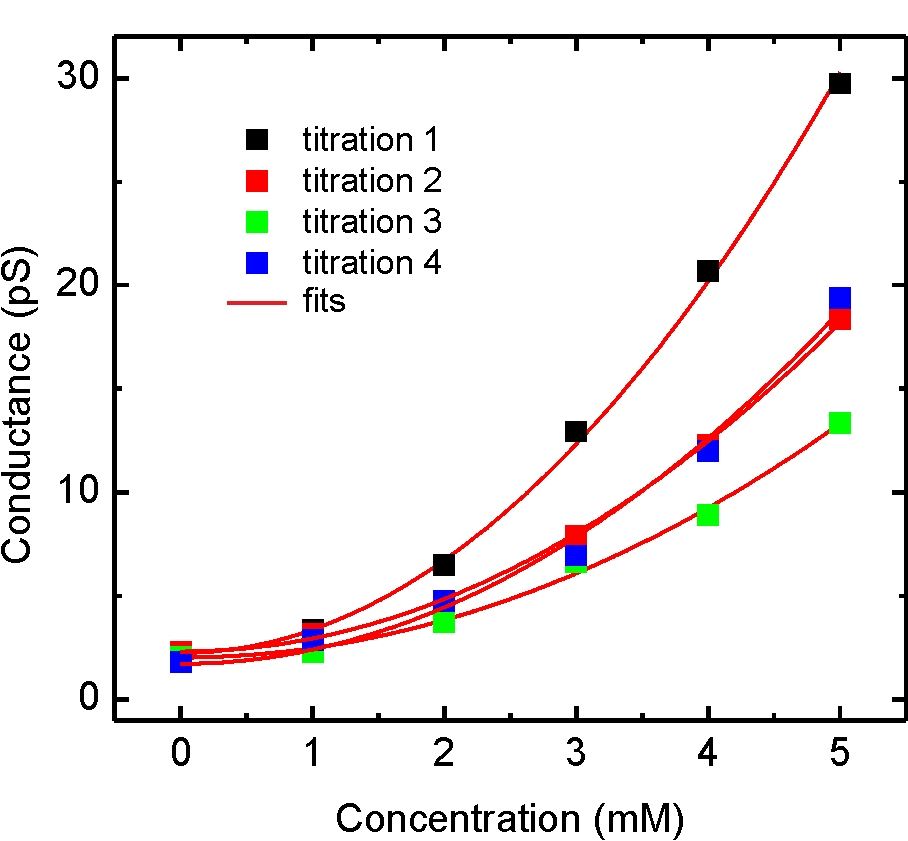


**Figure 4. The effect of indole on the ionic conductance, of an *E. coli* lipid membrane.** Four independent experiments were conducted to determine the effect of indole on membrane conductance in aqueous solution (100 mM KCl and pH 7.0 in PB). The dataset from each experiment was fitted to a quadratic function (eq.2).


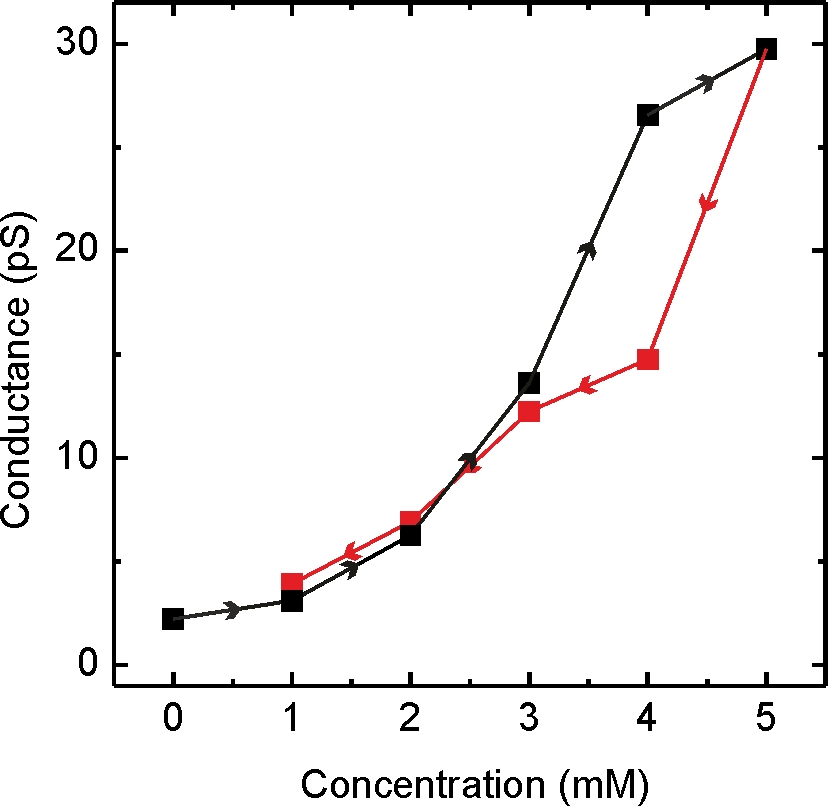


**Figure 5. Reversibility of the effect of indole on the ionic conductance of an *E. coli* lipid membrane** The change in conductance of the lipid membrane (aqueous solution; 100 mM KCl and pH 7.0 in PB) as the indole concentration increased from 0 to 5 mM (black line) was compared with the change as the concentration was reduced from 5 to 1 mM (red line). Arrows show the sense in which the concentration was changed.


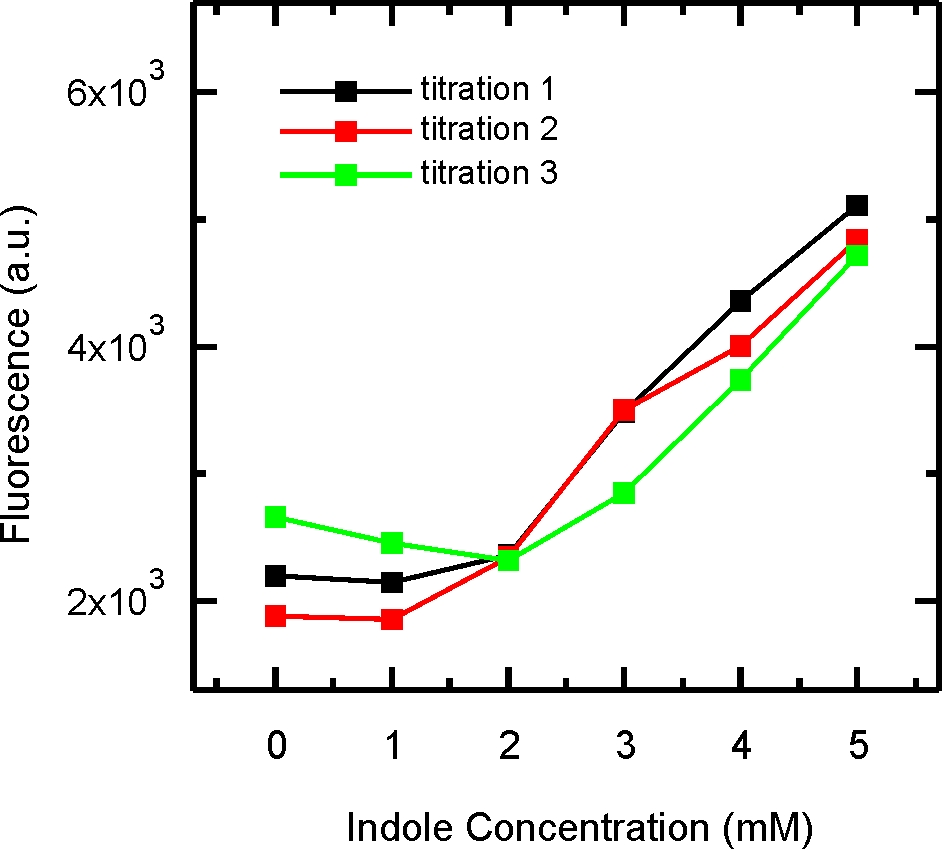


**Figure S6. Indole depolarises the *E. coli* membrane in a concentration-dependent manner.** In three independent experiments *E. coli* W3110ΔtnaA was treated with the lipophilic dye Oxonol VI in the presence of indole (0-5 mM). Cells were observed for fluorescence by flow cytometry and the median value of each sample was plotted against indole concentration.

**Supplementary Movies**

**Video S1.** The movie shows the oscillation of MinD between the poles of *E. coli* in the absence of indole. Fluorescent protein was expressed from plasmid pFX9 (Plac-GFP-MinD MinE) in all three supplementary movies. The movie represents 261.2 s (real time) and is composed of 76 frames. The scale bar represents 5µm.

**Video S2.** The movie shows the absence of MinD oscillation between the poles of the *E. coli* in the presence of 5mM indole. The movie represents 130.5 s (real time) and is composed of 34 frames. The scale bar represents 5µm.

**Video S3.** The movie, like supplementary movie 2, shows the absence of MinD oscillation between the poles of the *E. coli* in the presence of 5mM indole. The movie represents 87 s (real time) and is composed of 23 frames. The scale bar represents 5µm.
